# Historical Perspective of the G Protein-Coupled Receptor Kinase Family

**DOI:** 10.3390/cells10030555

**Published:** 2021-03-04

**Authors:** Jeffrey L. Benovic

**Affiliations:** Department of Biochemistry and Molecular Biology, Sidney Kimmel Medical College, Thomas Jefferson University, Philadelphia, PA 19107, USA; jeffrey.benovic@jefferson.edu

**Keywords:** arrestins, GPCR, GRK, phosphorylation, signaling

## Abstract

Agonist activation of G protein-coupled receptors promotes sequential interaction of the receptor with heterotrimeric G proteins, G protein-coupled receptor kinases (GRKs), and arrestins. GRKs play a central role in mediating the switch from G protein to arrestin interaction and thereby control processes such as receptor desensitization and trafficking and arrestin-mediated signaling. In this review, I provide a historical perspective on some of the early studies that identified the family of GRKs with a primary focus on the non-visual GRKs. These studies included identification, purification, and cloning of the β-adrenergic receptor kinase in the mid- to late-1980s and subsequent cloning and characterization of additional members of the GRK family. This helped to lay the groundwork for ensuing work focused on understanding the structure and function of these important enzymes.

## 1. Introduction

G protein-coupled receptors (GPCRs) are the largest family of membrane-localized proteins in mammals and function to enable cells to transmit extracellular stimuli such as hormones, chemokines, ions, peptides, and sensory stimuli into intracellular functional changes [1]. GPCRs primarily mediate their effects via the ability to interact in an activation-dependent manner with three protein families: heterotrimeric G proteins, GPCR kinases (GRKs), and arrestins. Initial work by the Rodbell and Gilman laboratories led to the discovery of the heterotrimeric G proteins [2], whereas studies in retinal rod cells by several groups led to the initial discovery of GRKs and arrestins. Subsequent work in the Lefkowitz and Caron laboratories led to the initial identification of non-visual GRKs and arrestins. In this review, I provide a historical overview of the initial discovery and characterization of the GRKs largely from my own personal perspective. This was the primary focus of my research in the Lefkowitz lab in the 1980s as well as a major focus of studies in my own laboratory starting in 1989.

## 2. Discovery of Rhodopsin Kinase

The history of the GRKs began in the 1970s with research in the visual system. Initial work by three groups demonstrated light-dependent phosphorylation of rhodopsin in rod membranes [3,4,5], whereas additional studies were able to extract a kinase from rod membranes that could phosphorylate photobleached rhodopsin [6]. The kinase was initially named opsin kinase [6] and later rhodopsin kinase (now called GRK1), and it was subsequently purified and found to specifically phosphorylate light-activated rhodopsin [7,8]. The latter purification involved salt extraction of rod outer segments followed by sequential fractionation on DE-cellulose 52 and hydroxyapatite resulting in a 1000-fold overall purification and recovery of 50 μg of purified kinase from 400 bovine retinas [8]. Properties of rhodopsin kinase include a molecular weight of 67–70 kDa, specificity for phosphorylation of photoactivated rhodopsin, and insensitivity to various second messengers, including cAMP, cGMP, and inositol trisphosphate.

## 3. Discovery of the β-Adrenergic Receptor Kinase (βARK)

Studies in the Lefkowitz laboratory during the late 1970s and early 1980s were focused on understanding the mechanisms involved in the loss of responsiveness of β-adrenergic receptor (βAR) signaling following prolonged stimulation with a β-agonist, a process that was termed desensitization or tachyphylaxis. These studies revealed that the βAR underwent a mobility shift on gel electrophoresis following agonist treatment of cells and demonstrated that this shift was due to receptor phosphorylation [9,10]. Additional studies established that at least some of this phosphorylation was due to the cAMP-dependent protein kinase (PKA) [11]. Intertwined with our understanding of this process were studies focused on purification of the β-adrenergic receptor, initially from frog [12] and turkey [13] erythrocytes followed by mammalian tissues such as hamster, rat, and guinea pig lung [14]. The purified hamster β_2_-adrenergic receptor (β_2_AR) was then fed into three projects: (1) one to reconstitute the receptor into phospholipid vesicles to determine if the purified receptor could directly activate the purified stimulatory heterotrimeric G protein (G_s_ or N_s_) in an agonist-dependent manner [15,16]; (2) one to obtain amino acid sequence to use in cloning a cDNA for the β_2_AR [17]; and (3) one to better understand the process of receptor desensitization. In regard to the desensitization efforts, in vitro studies demonstrated that PKA could directly phosphorylate the purified β_2_AR to a stoichiometry of 2 mol phosphate/mol receptor and that this phosphorylation directly inhibited receptor coupling to G_s_, although only 25% inhibition was observed in these initial studies [18]. Subsequent studies found that PKA phosphorylation of the receptor inhibited β_2_AR-G_s_ coupling by 60% at physiological concentrations of Mg^2+^ [19]. Thus, these studies identified a mechanism of feedback regulation that involved phosphorylation of the β_2_AR by PKA, the protein kinase activated by the βAR-signaling pathway. While this feedback regulation of the β_2_AR by PKA was termed heterologous or agonist-independent desensitization, one interesting aspect of the in vitro work was that the β_2_AR was found to be a better substrate for PKA in the presence of the agonist [18], suggesting that there may be an agonist-dependent or homologous component to this feedback regulation. To my knowledge, this aspect of the regulation has never been further explored.

**Figure 1:**
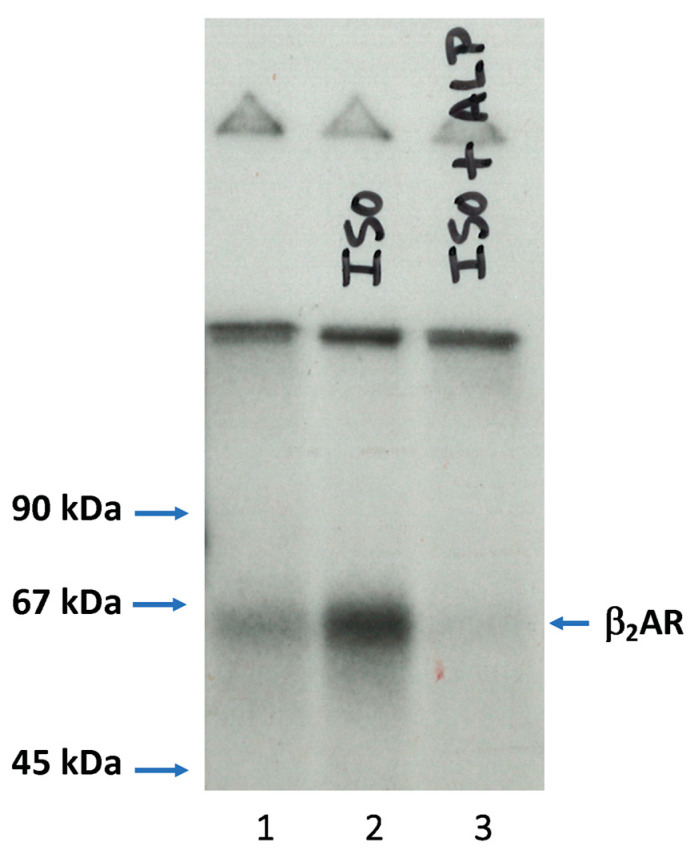
S49 kin^−^ cell extract phosphorylation of purified β_2_AR. S49 lymphoma kin^−^ cells were lysed, centrifuged, and the supernatant was incubated with MgCl_2_, radiolabeled ATP, purified hamster lung β_2_AR reconstituted in phospholipid vesicles, and either no ligand (lane 1), 10 μM isoproterenol (ISO) (lane 2), or 10 μM ISO plus 20 μM alprenolol (ALP) (lane 3) for 30 min at 30 °C. The samples were centrifuged, and the pellets were washed, solubilized, and the β_2_AR was purified on a small alprenolol affinity column and then run on SDS-PAGE and exposed to film.

While a role for PKA phosphorylation of the β_2_AR was evident from these early studies, additional efforts revealed that the β_2_AR could also be phosphorylated in an agonist-dependent manner in S49 lymphoma cell lines that could not activate PKA either because they lacked the ability to produce cAMP (cyc^−^ cells) or they lacked the PKA catalytic subunit (kin^−^ cells) [20]. This observation led to a search for the enzyme that phosphorylated the β_2_AR in an agonist-dependent manner and ultimately resulted in the identification of the β-adrenergic receptor kinase or βARK (now called GRK2) [21]. A critical aspect of this work was the ability to use the purified β_2_AR as a substrate, which facilitated the demonstration that this was an agonist-dependent process. Indeed, the first successful experiment testing the ability of an S49 kin^−^ cell extract to phosphorylate the purified receptor revealed that phosphorylation was increased in the presence of the β-agonist isoproterenol and that the agonist effect was blocked by the addition of the β-antagonist alprenolol (Figure 1).

βARK had clear similarities with rhodopsin kinase in that both enzymes phosphorylated the active form of the receptor. These studies were contiguous with the cloning of a cDNA for the β_2_AR and, taken together, raised interesting questions about the similarities between phototransduction through rhodopsin and hormonal signaling through the β_2_AR [22]. Indeed, additional studies demonstrated that βARK could phosphorylate light-activated rhodopsin whereas rhodopsin kinase could phosphorylate the agonist-occupied β_2_AR, albeit with each kinase preferring its endogenous substrate [23]. An additional series of studies suggested that βARK might have broad specificity for GPCRs since activation of the prostaglandin E_1_ [24] or somatostatin [25] receptors promoted βARK translocation from the cytosol to the plasma membrane. Moreover, βARK was also able to directly phosphorylate purified α_2_-adrenergic receptors in an agonist-dependent manner [26]. Taken together, these early studies suggested that βARK had a broad specificity and likely regulated the agonist-dependent phosphorylation of many GPCRs.

The next step in this work involved developing strategies to purify βARK. This initially involved searching for a tissue that had a high level of βARK expression with minimal degradation of the enzyme. Analysis of a number of tissues identified bovine cerebral cortex as a good source of the βARK activity (i.e., the ability to phosphorylate purified β_2_AR in an agonist-dependent manner), whereas tissues such as lung, spleen, liver, and heart had much lower levels of activity [27]. To purify the enzyme, the tissue was homogenized, centrifuged, and an ammonium sulfate cut of the soluble fraction was then purified through Ultrogel AcA34, DEAE-Sephacel, CM-Fractogel, and hydroxylapatite columns. This resulted in an overall purification of 20,000-fold and recovery of 12 μg of purified kinase from 100 g of tissue. Purified βARK was 80 kDa, it phosphorylated the agonist-occupied β_2_AR with a K_m_ of 0.25 μM, and it had many properties similar to rhodopsin kinase.

**Figure 2:**
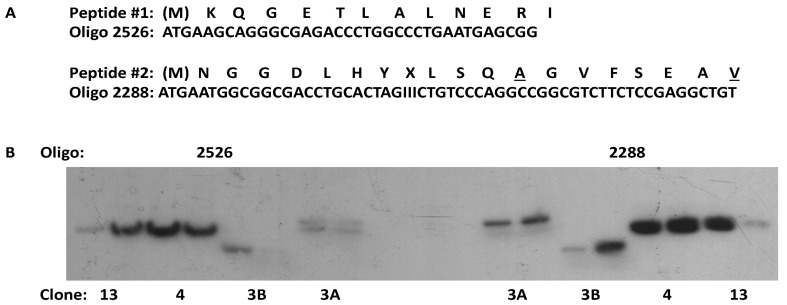
Cloning of a β-adrenergic receptor kinase (βARK) cDNA from a bovine brain cDNA library. One nmol of purified βARK was treated with CNBr, and the resulting peptides were separated and sequenced. A total of eight distinct peptide sequences were identified, with two being used in the design of oligonucleotide probes for cloning. (**A**) Amino acid sequence of two peptides that were consistently observed in multiple βARK preparations and the sequences of the oligonucleotide probes that were used to hybridize with cDNA libraries. The underlined residues in peptide #2 were found to be incorrect. (**B**) A Southern blot of several clones (3A, 3B, 4, and 13) isolated from the bovine brain cDNA library using radiolabeled oligo 2526 derived from peptide #1. Two colonies from each clone were digested with Eco RI, transferred to nitrocellulose, and probed with radiolabeled oligos derived from peptide #1 (2526) or peptide #2 (2288). The clones were sequenced, and clones 3A and 4 were found to contain a complete open reading frame. I—inosine.

Once βARK was purified, the next major goal was to clone a cDNA that encoded the protein. While we were able to purify βARK in adequate quantities to obtain amino acid sequence from cyanogen bromide-derived peptides, the subsequent cloning turned out to be an arduous process since all of the commercial cDNA libraries that were screened turned up nothing useful—always some positive clones on screening but never any sequences that matched the sequence of the kinase. So, what was wrong? Was it the amino acid sequence obtained from the purified βARK preparations? Maybe, but three different βARK preparations yielded consistent results, so we were confident in the amino acid sequences we were using to generate the radiolabeled oligonucleotides for screening (Figure 2A). Was it the cDNA libraries we were using? This certainly seemed to be a possibility since the libraries were mainly commercially obtained, and we were searching for something of low abundance. I attempted to address this issue by making my own cDNA library, but this again yielded nothing useful. We then turned to Dr. Richard Dixon at Merck Sharp and Dohme Research Laboratories for help since he had played a major role in the cloning of the β_2_AR [17]. Following a phone call from Bob Lefkowitz, Dr. Dixon provided us with a bovine brain cDNA library that he had personally created. The library was screened in February 1989, and we knew that we had bona fide βARK clones that hybridized with multiple probes within 10 days (Figure 2B). I had been screening cDNA libraries for two years at that point, so the level of excitement with the results is hard to describe but certainly in my top 10 list. Several overlapping clones were quickly sequenced and revealed an open reading frame of 2067 bp encoding a 689 amino acid protein [28]. Evidence supporting that the clone encoded βARK included the demonstration that expression in COS-7 cells yielded a protein that could phosphorylate the β_2_AR in an agonist-dependent manner. Moreover, a Southern blot of Hind III- or Sac I-digested bovine genomic DNA identified several low-stringency hybridizing bands, suggesting the presence of βARK-related genes [28].

## 4. The GRK Family

Once βARK was cloned, the next major goal was to identify additional members of the family. βARK2 (now called GRK3) was the next member cloned and resulted from low-stringency hybridization studies using the βARK cDNA to probe a bovine brain cDNA library [29]. βARK2 was 688 amino acids and had 85% amino acid identity to βARK, with the kinase catalytic domain being 95% identical (Table 1). βARK2 had lower activity against the β_2_AR and rhodopsin as substrates and tended to be expressed at a lower level in most tissues compared to βARK.

Rhodopsin kinase was the next GRK to be cloned via collaborative efforts between the Palczewski and Lefkowitz laboratories. This involved obtaining amino acid sequences of the purified protein to generate oligonucleotide probes that were used to screen a bovine retina cDNA library. This yielded clones encoding a 561 amino acid protein with 33.5% overall identity to βARK [30] (Table 1). Interestingly, the C-terminal domain of rhodopsin kinase was much shorter than that of βARK and ended with a CAAX motif suggesting that the protein may be prenylated. Indeed, subsequent studies showed that rhodopsin kinase is farnesylated and that this modification is important for full kinase activity [31]. The fourth member of the family to be cloned was called IT11 (interesting transcript 11, now called GRK4) and was fortuitously cloned as a result of its location near the Huntington’s disease locus [32]. The original IT11 clone encoded a 500 amino acid protein and had 48% overall identity to rhodopsin kinase and 36% to βARK (Table 1). A subsequent study showed that there are four splice variants of GRK4, that expression was primarily limited to the testes, and that the protein was palmitoylated [33].

Efforts from a number of laboratories ultimately led to the cloning and characterization of GRK5 and GRK6. GRK5 was initially cloned by the Benovic laboratory using degenerate oligonucleotides derived from unique GRK regions to amplify human heart cDNA using the polymerase chain reaction (PCR) [34]. GRK5 was 590 amino acids and had the highest amino acid homology with IT11, followed by rhodopsin kinase and βARK (Table 1). GRK5 expression was highest in heart and lung and lowest in brain, liver, and kidney. GRK5 was also cloned from a taste cell cDNA library [35]. It is worth noting that while the first two members of the GRK family were named after their substrate (rhodopsin or β_2_AR), the initial study identifying GRK5 recommended that the nomenclature be standardized [34]. The recommendation in the originally submitted manuscript was to use the GPRK nomenclature that had been used for two *Drosophila* members of the G protein-coupled receptor kinase family that had been identified, GPRK1 and GPRK2 [36]. However, the reviewer of the manuscript recommended the use of GRK instead of GPRK, so this ultimately led to the current naming of the family. The kinases were then named based on their order of discovery with GRK1 for rhodopsin kinase, GRK2 for βARK, GRK3 for βARK2, and GRK4 for IT11.

Additional efforts in the Benovic laboratory used the GRK2 and GRK3 cDNAs to screen a human heart cDNA library by low-stringency hybridization. This yielded the sixth member of the family (GRK6), a 576 amino acid protein with the highest identity to GRK5 and GRK4 [37]. GRK6 had an expression pattern similar to GRK2 with the highest levels in brain and skeletal muscle > heart, lung and kidney > liver. Initial functional studies suggested that GRK6 activity was significantly lower than GRK2, at least using rhodopsin and the β_2_AR as substrates. GRK6 was also cloned from cDNA prepared from human neutrophils using PCR amplification [38], although this clone appears to be a splice variant since it lacks the region that encodes the N-terminal 32 amino acids found in the GRK6 cloned from the human heart cDNA library. The final mammalian member of the GRK family to be cloned was GRK7 [39,40]. GRK7 cloned from mammalian species by RT-PCR was 548 amino acids with expression in cones [39], whereas cloning from medaka yielded a 563 amino acid protein containing a putative C-terminal site for geranylgeranylation [40]. GRK7 is most homologous to GRK1, GRK4, GRK5, and GRK6 (all 46–48% identity) with lower homology to GRK2 and GRK3 (31–32%).

## 5. Role of GRKs in Desensitization

During the course of the early work on GRKs, another protein that contributes to the regulation of GPCR function was identified. This protein was initially described as a 48 kDa protein that interacted with photoreceptor membranes in a light-dependent manner [41]. This 48 kDa protein was found to be identical to another rod cell protein called S-antigen and was shown to bind to phosphorylated rhodopsin and inhibit rod cGMP phosphodiesterase activity [42,43,44]. The protein was ultimately named arrestin based on its ability to bind to light-activated rhodopsin and arrest or turn-off phototransduction. A key feature of arrestin was its ability to bind to light-activated rhodopsin that had been phosphorylated by rhodopsin kinase [44]. While similarities between the regulation of phototransduction and hormonal signaling were starting to be established in the mid-1980s [21,22], these similarities were extended by efforts supporting a role for an arrestin in desensitizing β_2_AR signaling [45]. These studies found that crude βARK preparations had the ability to phosphorylate purified β_2_AR and effectively inhibit receptor interaction with purified G_s_. β_2_AR phosphorylation by more highly purified βARK preparations, however, had a modest effect on receptor-G_s_ coupling. This suggested the loss of a desensitization-factor during the βARK purification, and we were able to demonstrate that purified visual arrestin could inhibit β_2_AR interaction with G_s_ in a phosphorylation-dependent manner, albeit at very high stoichiometries [45]. This suggested that the desensitization factor that was lost during the βARK purification was a non-visual arrestin, although our attempts to isolate this factor were unsuccessful. Nevertheless, these efforts ultimately led to the cloning of a non-visual arrestin termed β-arrestin (now called β-arrestin-1 or arrestin-2) that could specifically bind to βARK-phosphorylated β_2_AR and inhibit receptor interaction with G_s_ [46]. Thus, these early studies found that GRKs play a central role in promoting arrestin binding to agonist-activated GPCRs and function to turn off receptor interaction with heterotrimeric G proteins, a process termed agonist-specific or homologous desensitization. We now know that there are four arrestins, two visual and two non-visual, that coordinate with GRKs to desensitize GPCR signaling through G proteins [47,48]. Moreover, the two non-visual β-arrestins also play important roles in GPCR trafficking [49,50] as well as arrestin-mediated signaling [51,52], although mechanistic details of these processes remain to be fully defined.

## 6. Summary

While the studies described above highlight some of the initial work identifying the GRK family, we now know much more about the role of GRKs in regulating GPCR function, a topic that has been extensively reviewed over the years [47,48,53,54,55,56,57,58]. What is left to do? We know a lot about GRKs and GRK activation from structural studies [59,60,61,62,63,64,65,66] and have some mechanistic insight on GRK-GPCR interaction [67,68]. For example, GRK5 binding to the β_2_AR promotes disruption of an ionic lock between the catalytic and regulator of G protein signaling homology (RH) domains that normally helps to maintain the kinase in a basal state [67], whereas a similar conformational change is observed when calmodulin binds to GRK5, leading to disruption of catalytic-RH domain interaction and kinase activation [69]. However, we still do not know what determines the specificity of GRK-GPCR interaction. We know that GRKs are activated by binding to GPCRs [70,71], but we do not know if this activation solely enhances GPCR phosphorylation or whether it also enhances the phosphorylation of additional substrates. We know that GRKs can phosphorylate many proteins other than GPCRs [55,56,57,72], but there have been no broad-based proteomic studies to broadly define the GRK phospho-proteome. GRKs have also been implicated in the barcode hypothesis that proposes that different sites of GPCR phosphorylation will induce distinct conformations and functions of bound arrestin [73,74], but we know little about the link between specific sites of phosphorylation, the GRK and arrestin involved, and the functional consequences. We also know that GRKs are potential targets in the treatment of several diseases, including cardiovascular disease, metabolic and neurological disorders, and cancer [55,56,57], but much remains to be learned about the best strategies to target specific GRKs and the side effects that might occur in doing so. We have learned much about GRKs since their initial discovery some 40 plus years ago, but there seems to be plenty left to do.

## Figures and Tables

**Table 1:** Amino acid homology of human G protein-coupled receptor kinases (GRKs).

	GRK1	GRK2	GRK3	GRK4	GRK5	GRK6	GRK7
GRK1	100	52	52	66	69	68	66
GRK2	33.4	100	92	54	54	55	53
GRK3	33.5	84.0	100	53	53	55	53
GRK4	47.7	36.4	36.5	100	81	79	65
GRK5	47.8	37.2	37.1	68.0	100	84	65
GRK6	48.2	38.7	38.9	66.6	72.3	100	66
GRK7	47.6	31.2	32.2	47.9	45.9	46.5	100

Percent amino acid identity (in black) or similarity (in red) of human GRKs. Pairwise comparisons were conducted using sequences from human GRK1 (563 amino acids, Q15835 from UniProtKB), GRK2 (689 amino acids, P25098), GRK3 (688 amino acids, P35626), GRK4α (578 amino acids, P32298), GRK5 (590 amino acids, P34947), GRK6A (576 amino acids, P43250), and GRK7 (553 amino acids, Q8WTQ7) using BlastP at blast.ncbi.nlm.nih.gov.

## Data Availability

No new data were created or analyzed in this study.
